# Recognizing the Contribution of Data: The Use of Companion Articles To Highlight Novel Resources in Microbiology Research

**DOI:** 10.1128/aem.02153-22

**Published:** 2023-01-19

**Authors:** Jack A. Gilbert, Irene L. G. Newton, Gemma Reguera

**Affiliations:** a University of California, San Diego School of Medicine, La Jolla, California, USA; b Department of Biology, Indiana University, Bloomington, Indiana, USA; c Department of Microbiology and Molecular Genetics, Michigan State University, East Lansing, Michigan, USA

## Abstract

As part of society-wide efforts to promote open access in science, the American Society for Microbiology journals are piloting the publication of companion articles highlighting rigorous data resources. The simultaneous publication of original research and data resource articles will increase awareness of, and access to, verified data sets that are critical to scientific progress. Companion articles in *Microbiology Resource Announcements* and two research journals, *mSystems* and *Applied and Environmental Microbiology*, will serve as an initial experiment to promote open and reproducible science.

## EDITORIAL

The impact, reproducibility, and rigor of experimental science are predicated on how easy it is to access, reanalyze, and interpret the scientific data that support findings published in primary research articles. Equally important is access to tools developed to generate or analyze scientific data. The “Open Science” framework aspires to disseminate research products, findings, tools, and interpretations in a format that is accessible to all levels of society (1, 2). Despite these efforts, scientists continue to face great hurdles to access and reanalyze published data or to use published tools effectively. Such challenges significantly impede reproducibility, reuse, and collaboration. Open access and data sharing policies help overcome some of these challenges. Indeed, primary research articles that disseminate initial study findings or benchmark new tools and techniques are typically required to include a data or tool accessibility paragraph. However, no consistent attempt is made to ensure that these resources are truly accessible and reusable.

*Microbiology Resource Announcements* (*MRA*) was envisioned as a platform through which microbiologists could publish tools, genetic resources, “omics” data sets, databases, and workflows—any resource useful to the microbiology community at large. Throughout the years since its launch, the journal’s editor in chief and senior editors have further refined *MRA*’s mission to ensure that resources are easily accessible, reproducible, and clearly annotated for the microbiology community. An *MRA* announcement allows authors to provide clear methodological information about how the data were generated and analyzed and also provide open access links to the raw data. These pieces of information go hand in hand. For example, without understanding how an experiment was designed and run on an Illumina MiSeq system, it can be difficult, if not impossible, to reuse the raw reads published in the NCBI Sequence Read Archive.

Evaluation and validation of scientific resources through *MRA* add value. The *MRA* review process is much like that of any journal, but it is driven by the reviewers’ focus on the resource’s availability and utility. Careful evaluation of the resource ensures that the authors’ description in their *MRA* manuscript matches the accession numbers and DOIs provided. Specific details, such as the parameters used for all bioinformatic software involved in generating the resource and the provision of the material in question, are required by *MRA.* These types of details may be easily overlooked during the review of primary publications, in which reviewers often focus on the *interpretation* of the scientific results and not necessarily the nitty gritty of the resource itself. *MRA* provides a “verification stamp” on the resource; it has been thoroughly checked by two experts in the field and gone through extensive review to ensure that the resource is as open—and useful—as possible to the community.

We are excited by the potential of *MRA* companion articles to increase the rigor, accessibility, and reusability of the resources being leveraged in your primary research articles. In this pilot endeavor, *MRA* joins forces with two ASM journals, *Applied and Environmental Microbiology* (*AEM*) and *mSystems*, that are committed to best practices in data sharing and accessibility. This partnership will allow authors to submit original research articles to *AEM* and *mSystems* with *MRA* companion articles focused on expanded, rigorous analysis of data resources reported in the primary article. A companion article may have an authorship order different from that of the primary publication as needed to showcase the contributions of other members of a research team and support their career progress. Importantly, the *MRA* companion article is peer reviewed under the highest journal standards to provide much-needed verification and validation of data resources that are essential to repeat and further advance research. These resources—thanks to the evaluation at *MRA*—will provide collaborative resource pools to facilitate meta-analyses while also enhancing the utility of the primary source.

The launch of this pilot program at ASM journals recognizes the value of data resources and addresses the need to curate these valuable resources in formats that are both accessible and usable for scientists. We will be evaluating how the community responds to this opportunity, and we will look for ways to improve its utility. We welcome your feedback! So, please, reach out and tell us how you think companion articles could be improved to realize their full potential.
